# Supplementary material for: Omicron BA.2 breakthrough infection enhances cross-neutralization of BA.2.12.1 and BA.4/BA.5
Source: Sci Immunol. 2022 Sep 20:eade2283. doi: 10.1126/sciimmunol.ade2283 (PMC9529054; doi:10.1126/sciimmunol.ade2283)
Supplement: Supplementary file 1 — Figs. S1 to S6 Tables S1 to S11 [file sciimmunol.ade2283_sm.pdf]

Science Immunology
AAAS

Supplementary Materials for

Omicron BA.2 breakthrough infection enhances cross-neutralization of BA.2.12.1 and BA.4/BA.5

Alexander Muik *et al.*

Corresponding author: Ugur Sahin, ugur.sahin@biontech.de

Sci. Immunol. , eade2283 (2022)
DOI: 10.1126/sciimmunol.ade2283

The PDF file includes:

Figs. S1 to S6
Tables S1 to S11

Other Supplementary Material for this manuscript includes the following:

MDAR Reproducibility Checklist

fig. S1

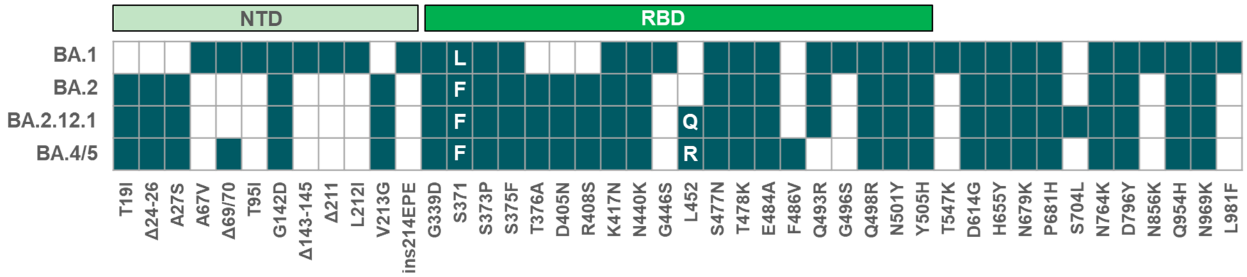

Fig. S1. Alterations of the spike glycoprotein amino acid sequence of SARS-CoV-2 Omicron sub-lineages.

Amino acid exchange and mutation type (substitutions, deletions, insertions) are indicated. White letters in boxes indicate the amino acid substitution per sub-lineage; Δ, deletion; ins, insertion; NTD, N-terminal domain; RBD, receptor-binding domain

fig. S2

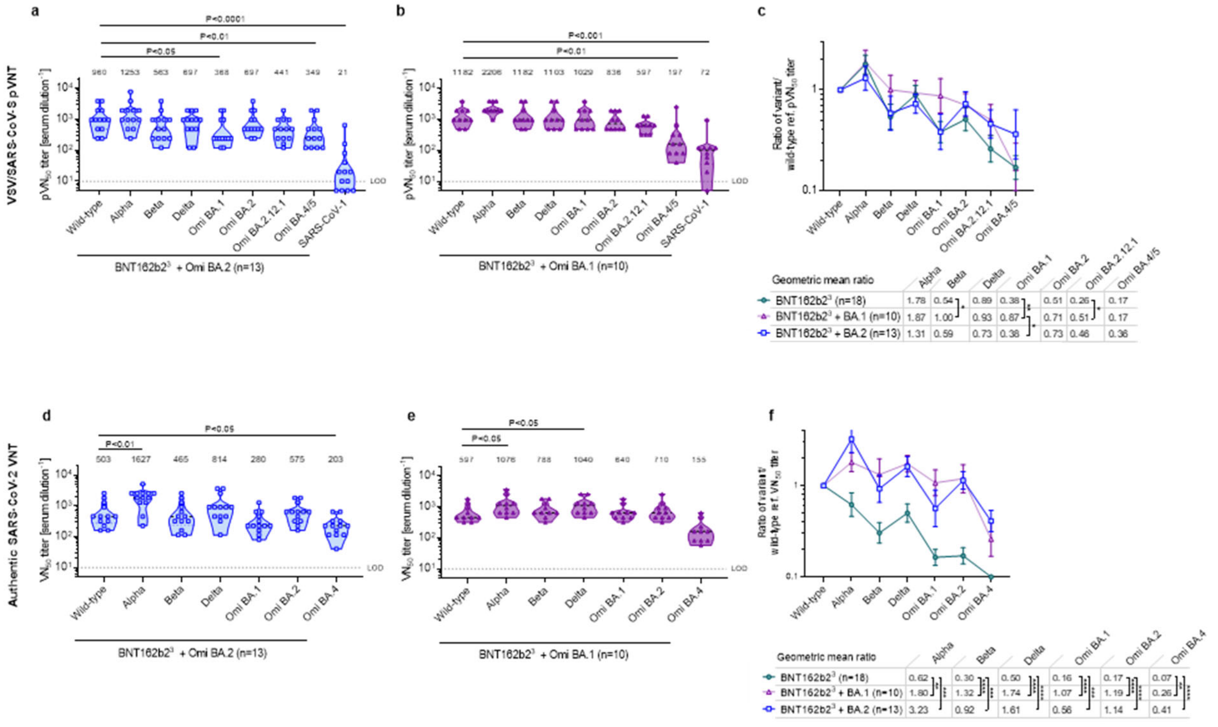

Fig. S2. Omicron BA.2 breakthrough infection of BNT162b2 triple-vaccinated individuals induces broad neutralization of VOCs including Omicron BA.4/BA.5.

Cohorts and serum sampling as described in Fig. 1. (a-b) 50% pseudovirus neutralization (pVN_{50}) geometric mean titers (GMTs) against the indicated SARS-CoV-2 variants of concern (VOCs) or SARS-CoV-1 pseudoviruses. Values above violin plots represent group GMTs. (c) The ratio of SARS-CoV-2 VOC pVN_{50} GMTs normalized against the wild-type strain pVN_{50} GMT. Geometric mean ratios for the Omicron BA.2 breakthrough infected cohort were compared to data previously published in Quandt et al. (*10*) for BNT162b2^3 and BNT162b2^3 + BA.1, except for newly generated BA.2.12.1 neutralization data. Group geometric mean ratios with 95% confidence intervals are shown. (d-e) 50% virus neutralization (VN_{50}) GMTs for BNT162b2^3 + BA.1 and BNT162b2^3 + BA.2. Values above violin plots represent group GMTs.

(f) The ratio of SARS-CoV-2 VOC GMTs normalized against the wild-type strain VN_{50} GMT. Serum was tested in duplicate. For titer values below the limit of detection (LOD), LOD/2 values are plotted. The non-parametric Friedman test with Dunn's multiple comparisons correction was used to compare the group GMT against the wild-type strain with group GMTs against the indicated variants and SARS-CoV-1 (a-b and d-e). Multiplicity-adjusted p values are shown. The non-parametric Kruskal-Wallis test with Dunn's multiple comparisons correction was used to compare the VOC GMT ratios between cohorts (c and f). ****, P<0.0001; ***, P<0.001; **, P<0.01; *, P<0.05.

fig. S3

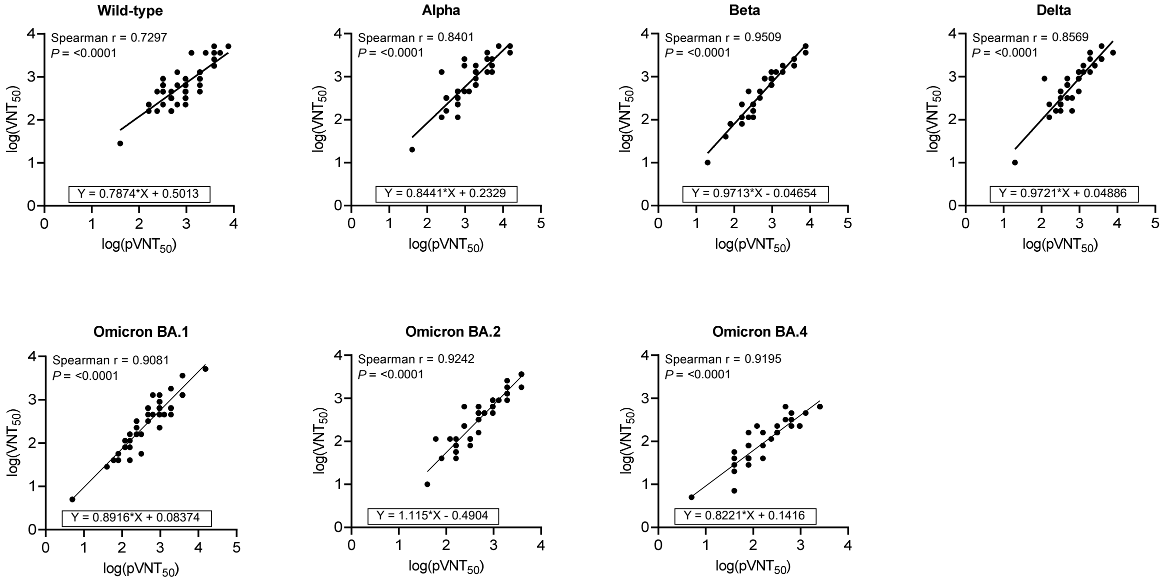

Fig. S3. 50% pseudovirus neutralization (pVN$_{50}$) correlates with 50% live SARS-CoV-2 neutralization (VN$_{50}$) titer data

Nonparametric Spearman correlation of VSV-SARS-CoV-2 pVN$_{50}$ with live SARS-CoV-2 VN$_{50}$ titers for n=51 serum samples drawn from SARS-CoV-2-naïve BNT162b2 triple-vaccinated individuals (BNT162b2^3; n=18) after the third dose, from triple mRNA vaccinated individuals with subsequent Omicron BA.1 breakthrough infection (mRNA-Vax^3 + BA.1; n=14) post-infection, and from triple mRNA vaccinated individuals with subsequent Omicron BA.2 breakthrough infection (mRNA-Vax^3 + BA.2; n=19) post-infection. Correlations are plotted per SARS-CoV-2 variant. Correlation coefficient r, two-tailed P values and the linear equation are given.

.

fig. S4

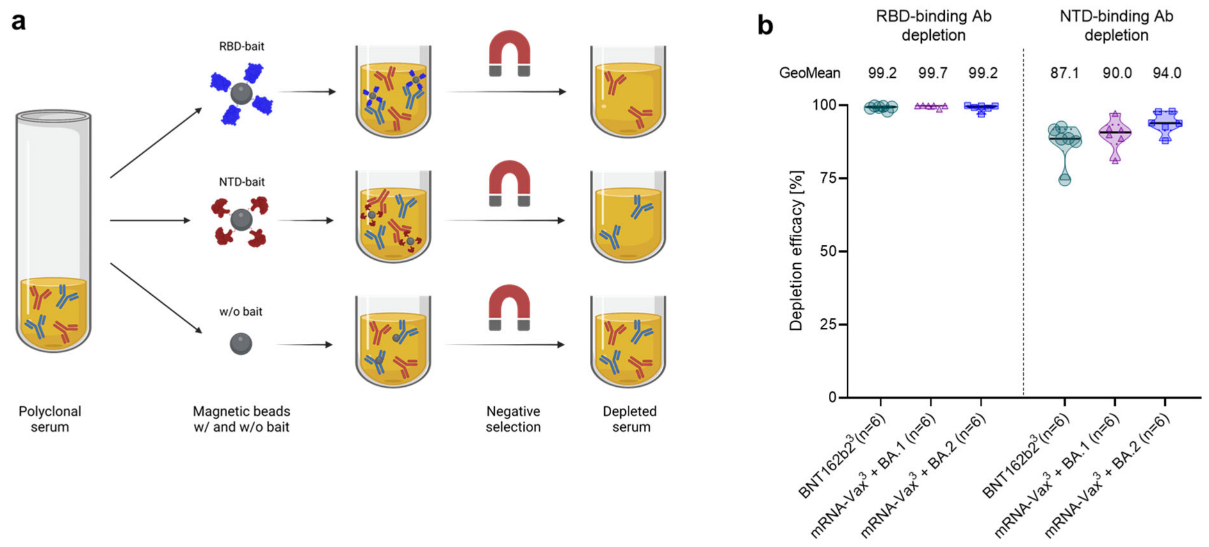

Fig. S4. RBD-binding and NTD-binding antibodies can be depleted from human serum

Serum was drawn from SARS-CoV-2-naïve BNT162b2 triple-vaccinated individuals (BNT162b23; n=6), and from triple mRNA vaccinated individuals with Omicron BA.1 (mRNA-Vax3 + BA.1; n=6) or Omicron BA.2 breakthrough infection (mRNA-Vax3 + BA.2; n=6). Magnetic bead technology was used for depleting serum of RBD- or NTD-binding antibodies, or for mock depleting. (a) Schematic of antibody depletion from serum. (b) The relative concentration of RBD-binding and NTD-binding antibodies was determined by a multiplexed electrochemiluminescence immunoassay. The relative decrease in antibody concentrations in depleted compared to mock-depleted sera are shown. Numbers above graph depict geometric mean reduction within groups.

Fig. S5

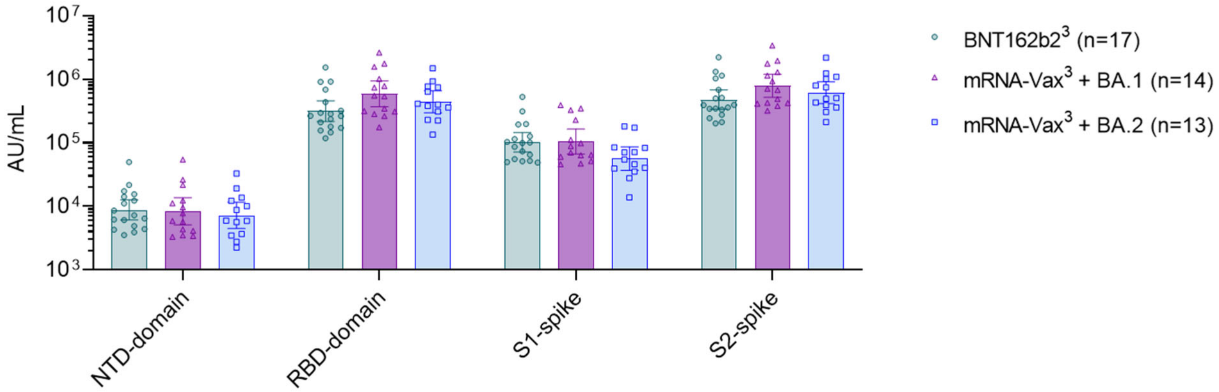

Fig. S5. Serum antibody levels targeting S glycoprotein domains are not significantly different between cohorts.

Cohorts and serum sampling as described in Fig. 1. Serum antibody levels targeting the NTD, RBD, S1 or S2 domains of the S glycoprotein were determined by electrochemiluminescence immunoassays. The non-parametric Kruskal-Wallis test with Dunn's multiple comparisons correction was used to compare concentrations of antibodies targeting each domain across cohorts. None of the comparisons reached statistical significance. AU, arbitrary units.

Fig. S6

a

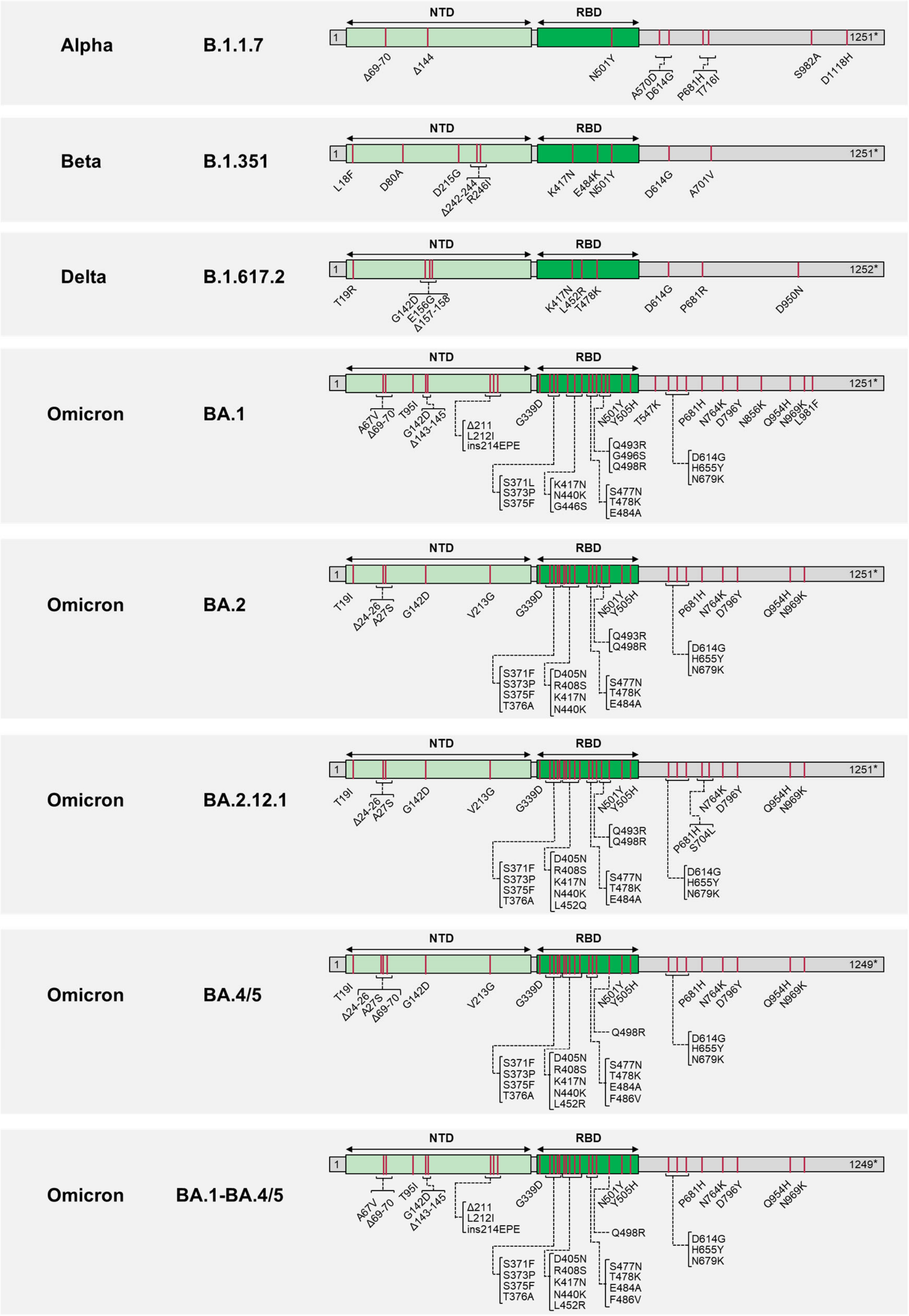
Alpha
B.1.1.7
NTD
RBD
1
1251*
Δ69-70
Δ144
N501Y
A570D
D614G
P681H
T716I
S982A
D1118H
Beta
B.1.351
NTD
RBD
1
1251*
L18F
D80A
D215G
Δ242-244
R246I
K417N
E484K
N501Y
D614G
A701V
Delta
B.1.617.2
NTD
RBD
1
1252*
T19R
G142D
E156G
Δ157-158
K417N
L452R
T478K
D614G
P681R
D950N
Omicron
BA.1
NTD
RBD
1
1251*
A67V
Δ69-70
T95I
G142D
Δ143-145
Δ211
L212I
ins214EPE
G339D
S371L
S373P
S375F
K417N
N440K
G446S
S477N
T478K
E484A
Q493R
G496S
Q498R
N501Y
Y505H
T547K
D614G
H655Y
N679K
P681H
N764K
D796Y
N856K
Q954H
N969K
L981F
Omicron
BA.2
NTD
RBD
1
1251*
T19I
Δ24-26
A27S
G142D
V213G
G339D
S371F
S373P
S375F
T376A
D405N
R408S
K417N
N440K
S477N
T478K
E484A
Q493R
Q498R
N501Y
Y505H
D614G
H655Y
N679K
P681H
N764K
D796Y
Q954H
N969K
Omicron
BA.2.12.1
NTD
RBD
1
1251*
T19I
Δ24-26
A27S
G142D
V213G
G339D
S371F
S373P
S375F
T376A
D405N
R408S
K417N
N440K
L452Q
S477N
T478K
E484A
Q493R
Q498R
N501Y
Y505H
D614G
H655Y
N679K
P681H
S704L
N764K
D796Y
Q954H
N969K
Omicron
BA.4/5
NTD
RBD
1
1249*
T19I
Δ24-26
A27S
Δ69-70
G142D
V213G
G339D
S371F
S373P
S375F
T376A
D405N
R408S
K417N
N440K
L452R
S477N
T478K
E484A
F486V
Q498R
N501Y
Y505H
D614G
H655Y
N679K
P681H
N764K
D796Y
Q954H
N969K
Omicron
BA.1-BA.4/5
NTD
RBD
1
1249*
A67V
Δ69-70
T95I
G142D
Δ143-145
Δ211
L212I
ins214EPE
G339D
S371F
S373P
S375F
T376A
D405N
R408S
K417N
N440K
L452R
S477N
T478K
E484A
F486V
Q498R
N501Y
Y505H
D614G
H655Y
N679K
P681H
N764K
D796Y
Q954H
N969K

b

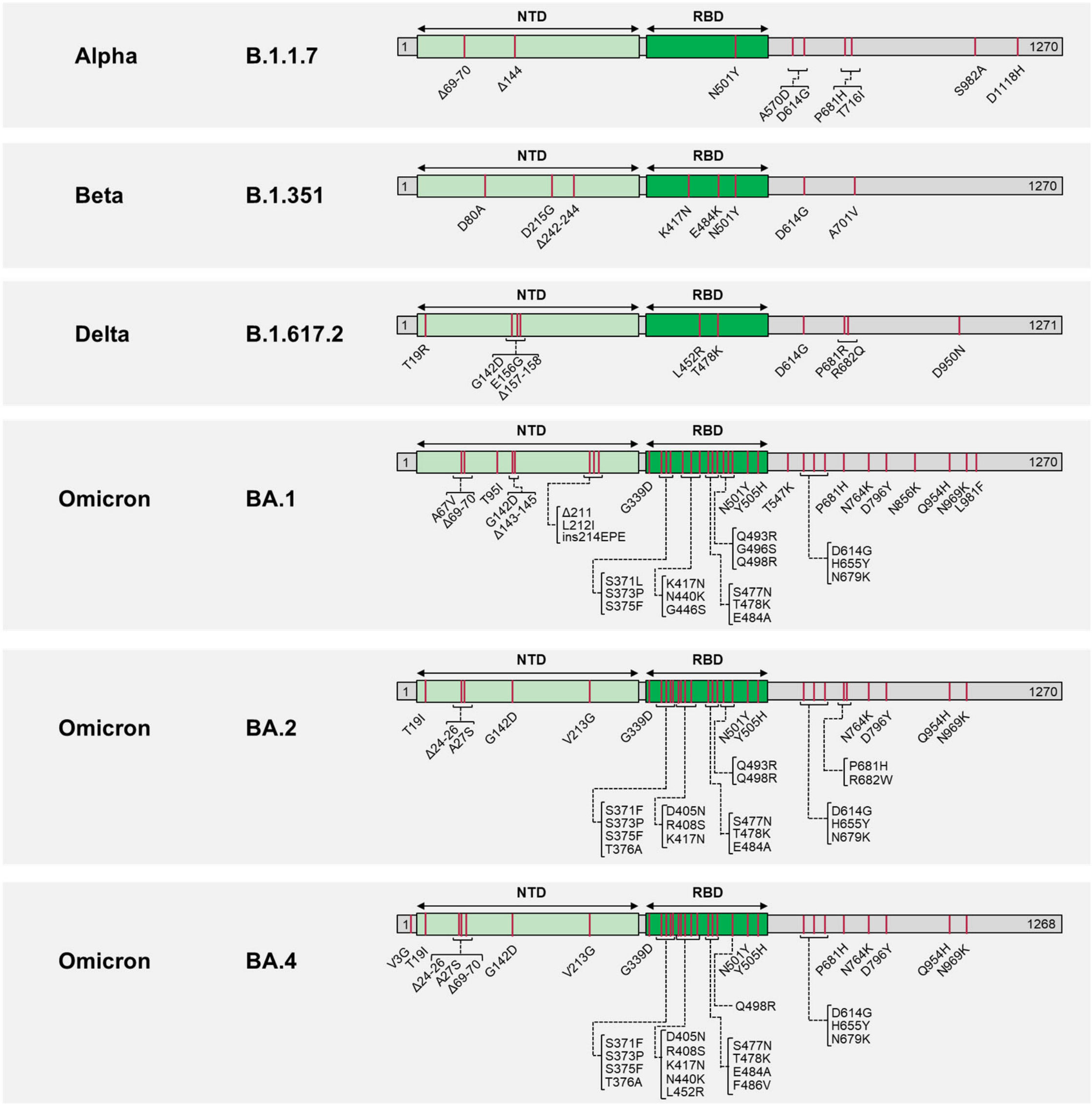

Fig. S6. Characterization of SARS-CoV-2 S glycoproteins used in the assays based on (a) VSV-SARS-CoV-2 variant pseudoviruses and (b) live authentic SARS-CoV-2.

The sequence of the Wuhan-Hu-1 isolate SARS-CoV-2 S glycoprotein (GenBank: QHD43416.1) was used as reference. Amino acid positions, amino acid descriptions (one letter code) and kind of alterations (substitutions, deletions, insertions) are indicated. NTD, N-terminal domain; RBD, Receptor-binding domain, Δ, deletion; ins, insertion; *, Cytoplasmic domain truncated for the C-terminal 19 amino acids.

Table S1. Vaccinated individuals analyzed for neutralizing antibody responses.

Characteristic	BNT162b2[3] (n=18)	mRNA-Vax[3] + BA.1 (n=14)	mRNA-Vax[3] + BA.2 (n=19)
Sex, n (%)			
Male	9 (50)	11 (79)	7 (37)
Female	9 (50)	3 (21)	12 (63)
Age, median (range)	38 (23-54)	32 (23-60)	30 (25-74)
Age group at vaccination, n (%)			
18-55 yrs	18 (100)	12 (86)	15 (79)
56-85 yrs	0 (0)	2 (14)	4 (21)
SARS-CoV-2 status, n (%)			
Positive	0 (0)	14 (100)#	19 (100)*
Negative	18 (100)†	0 (0)	0 (0)
Unknown	0 (0)	0 (0)	0 (0)
Interval, median (range)			
Days between D1/D2	‡	38 (20-92)	42 (15-43)
Days between D2/D3	202 (181-266)	192 (154-256)	184 (152-259)
Days until serum draw after D3	28 (26-30)	N/A	N/A
Days between last dose/infection	N/A	25 (3-112)	141 (36-200)
Days until serum draw after infection	N/A	43 (25-55)	43 (28-99)

N/A, not applicable; D, dose; yrs, years; n, number.

*, Individuals experienced SARS-CoV-2 breakthrough infections between March and May 2022, during which period the BA.2 lineage was dominant in Germany. BA.2 infection of two participants were confirmed by sequencing.

#, Omicron infection PCR-confirmed at time of recruitment to the research study. Individuals experienced SARS-CoV-2 breakthrough infections between November 2021 and January 2022, during which period the BA.1 lineage was dominant in Germany

†, No evidence of prior SARS-CoV-2 infection (based on COVID-19 symptoms/signs and SARS-CoV-2 PCR test)

‡, Participants received the primary 2-dose series of BNT162b2 vaccine as part of a governmental vaccination program and the interval between doses was not recorded

Table S2. Individuals triple vaccinated with mRNA COVID-19 vaccine and subsequently infected with Omicron BA.1 (mRNA-Vax³ + BA.1).

Participant ID	Age	Sex	Vaccination	Date positive test	Omicron subtype	Dose 1-2 interval (days)	Dose 2-3 interval (days)	Positive test after last vaccination (days)	Blood draw after positive test (days)	Severity (WHO grade)
14	32	f	BNT³	NOV2021	BA.1	24	243	64	55	1-2
15	32	m	BNT³	NOV2021	BA.1	20	233	66	53	1-2
16	28	m	BNT³	DEC2021	n/a	35	213	10	47	1-2
17	29	f	BNT³	DEC2021	BA.1	36	189	3	46	1-2
18	23	m	BNT³	DEC2021	n/a	42	159	27	44	1-2
19	31	m	BNT³	DEC2021	n/a	42	166	20	43	1-2
20	53	m	BNT³	JAN2022	n/a	39	194	22	25	1-2
21	50	f	BNT³	JAN2022	n/a	92	169	35	28	1-2
22	50	m	BNT³	JAN2022	n/a	42	169	44	31	1-2
23	60	m	BNT³	JAN2022	n/a	26	236	112	43	1-2
24	28	m	MOD²/BNT	DEC2021	n/a	28	252	22	40	1-2
25	32	m	MOD²/BNT	DEC2021	n/a	42	154	13	42	1-2
26	50	m	MOD²/BNT	JAN2022	n/a	28	256	45	31	1-2
27	60	m	MOD³	DEC2021	BA.1	42	172	3	35	1-2
**Median**	**32**	**N/A**	**N/A**	**N/A**	**N/A**	**38**	**192**	**25**	**43**	**N/A**

m, male; f, female; n/a, not available; N/A, not applicable

BNT, BioNTech/Pfizer BNT162b2; MOD, Moderna mRNA-1273; BNT³, BNT162b2 three-dose series; MOD², mRNA-1273 two-dose series; MOD³, mRNA-1273 three-dose series

Table S3. Individuals triple vaccinated with mRNA COVID-19 vaccine and subsequently infected with Omicron BA.2 (mRNA-Vax^3 + BA.2).

Participant ID	Age	Sex	Vaccination	Date positive test	Omicron subtype	Dose 1-2 interval (days)	Dose 2-3 interval (days)	Positive test after last vaccination (days)	Blood draw after positive test (days)	Severity (WHO grade)
1	42	f	BNT^3	APR2022	n/a	42	158	135	36	1-2
2	44	m	BNT^2/MOD	APR2022	n/a	42	164	129	36	1-2
3	28	f	MOD^2/BNT	MAR2022	n/a	42	163	36	99	1-2
4	26	f	BNT^3	APR2022	n/a	43	152	104	58	1-2
5	34	m	BNT^3	MAY2022	n/a	40	154	141	37	1-2
6	29	m	BNT^3	APR2022	n/a	42	165	89	63	1-2
7	57	f	MOD^2/BNT	APR2022	n/a	15	244	153	46	1-2
8	25	m	MOD/BNT^2	MAY2022	n/a	28	217	188	34	1-2
9	30	f	MOD^2/BNT	APR2022	n/a	42	195	129	66	1-2
10	28	f	BNT^3	MAY2022	n/a	21	224	64	39	1-2
11	29	f	BNT^3	APR2022	n/a	22	249	149	64	1-2
12	53	f	BNT^3	MAY2022	n/a	29	259	200	30	1-2
13	72	f	BNT^3	MAY2022	n/a	42	184	157	30	1-2
46	25	f	BNT^3	MAY2022	n/a	32	178	159	34	1-2
47	28	m	BNT^3	MAY2022	BA.2	42	175	199	28	1-2
48	74	m	BNT^3	MAR2022	n/a	42	173	122	92	1-2
49	63	f	BNT^3	MAR2022	n/a	42	184	109	94	1-2
50	27	f	MOD^2 /BNT	APR2022	n/a	35	212	146	71	1-2
51	49	f	BNT^3	MAY2022	BA.2	28	185	165	43	1-2
**Median**	**30**	**N/A**	**N/A**	**N/A**	**N/A**	**42**	**184**	**141**	**43**	**N/A**

m, male; f, female; n/a, not available; N/A, not applicable

BNT, BioNTech/Pfizer BNT162b2; MOD, Moderna mRNA-1273; BNT^2, BNT162b2 two-dose series; BNT^3, BNT162b2 three-dose series; MOD^2, mRNA-1273 two-dose series

Table S4. pVN_{50} values of sera collected from SARS-CoV-2-naïve triple-vaccinated individuals (BNT162b2[3])

Participant ID	pVN_{50}									
	Wild-type	Alpha	Beta	Delta	Omicron BA.1	Omicron BA.2	Omicron BA.2.12.1	Omicron BA.4/5	Omicron BA.1-BA.4/5	SARS-CoV-1
28	160	320	160	160	80	160	80	40	40	5
29	640	640	320	320	160	320	160	40	40	40
30	5120	5120	1280	2560	1280	1280	640	640	160	40
31	320	640	160	320	160	160	80	40	40	20
32	640	640	80	640	320	160	40	40	20	20
33	320	640	160	320	160	160	80	40	20	10
34	320	640	320	320	160	160	80	80	40	10
35	320	640	320	320	160	160	160	80	40	20
36	160	320	80	160	40	80	40	40	10	20
37	320	1280	160	320	160	60	160	80	40	20
38	1280	5120	640	1280	640	640	640	320	160	80
39	40	40	20	20	5	40	10	5	5	5
40	320	640	320	320	80	160	80	40	20	20
41	160	320	160	320	80	160	80	40	20	20
42	320	640	320	320	320	320	160	160	40	20
43	640	640	320	320	160	320	80	80	40	40
44	2560	5120	640	1280	640	640	320	320	160	80
45	320	640	160	640	160	320	80	80	80	20

Table S5. pVN_{50} values of sera collected from individuals with Omicron BA.1 breakthrough infection (mRNA-Vax^3 + BA.1)

Participant ID	pVN_{50}									
	Wild-type	Alpha	Beta	Delta	Omicron BA.1	Omicron BA.2	Omicron BA.2.12.1	Omicron BA.4/5	Omicron BA.1-BA.4/5	SARS-CoV-1
14	1920	3840	1920	1920	1920	960	640	320	80	120
15	960	1920	960	480	480	480	640	160	320	120
16	3840	3840	3840	3840	1920	1920	1280	640	640	960
17	960	1920	960	960	960	960	640	160	160	120
18	480	1920	960	480	480	480	640	40	40	20
19	1920	1920	960	960	960	1920	640	320	320	40
20	960	1920	960	960	1920	480	320	80	160	80
21	960	1920	480	960	480	480	320	80	80	5
22	480	960	480	480	480	480	320	80	40	60
23	1920	3840	3840	3840	3840	1920	1280	2560	1280	120
24	3840	15360	7680	7680	3840	3840	2560	2560	1280	120
25	7680	15360	7680	3840	15360	3840	2560	1280	640	480
26	480	240	60	240	60	120	40	40	10	30
27	1920	1920	960	1920	960	960	640	640	320	60

Table S6. pVN_{50} values of sera collected from individuals with Omicron BA.2 breakthrough infection (mRNA-Vax^3 + BA.2)

Participant ID	pVN_{50}									
	Wild-type	Alpha	Beta	Delta	Omicron BA.1	Omicron BA.2	Omicron BA.2.12.1	Omicron BA.4/5	Omicron BA.1-BA.4/5	SARS-CoV-1
1	480	1920	960	480	240	480	160	120	80	5
2	3840	7680	3840	3840	3840	3840	2560	960	160	40
3	480	960	240	240	240	240	240	120	40	10
4	240	480	120	120	120	240	120	120	10	5
5	1920	1920	480	960	240	480	480	240	80	20
6	480	480	240	480	240	480	240	240	80	5
7	960	960	480	960	960	960	480	480	160	5
8	960	1920	960	1920	960	960	960	480	320	20
9	1920	3840	960	1920	960	1920	1920	480	160	20
10	960	960	240	960	240	480	240	120	160	10
11	960	960	480	960	240	480	480	480	80	10
12	1920	1920	960	1920	240	1920	1920	1920	640	5
13	960	1920	960	960	960	960	480	1920	320	20
46	240	240	240	120	120	240	240	120	40	40
47	960	960	960	960	960	960	960	960	320	80
48	3840	7680	3840	1920	1920	3840	960	480	320	640
49	960	960	240	480	240	480	480	240	20	20
50	960	1920	960	480	960	960	960	960	160	160
51	3840	3840	1920	1920	1920	1920	960	480	320	160

Table S7. VN_{50} values of sera collected from SARS-CoV-2-naïve triple-vaccinated individuals (BNT162b2[3])

Participant ID	VN_{50}						
	Wild-type	Alpha	Beta	Delta	Omicron BA.1	Omicron BA.2	Omicron BA.4
28	226	320	80	226	40	40	28
29	640	320	226	226	160	80	28
30	3620	2560	1280	1810	453	905	226
31	226	113	226	160	40	80	28
32	226	453	80	160	57	40	20
33	640	453	113	226	80	57	40
34	453	226	226	453	80	80	28
35	640	320	226	226	160	113	40
36	160	160	80	113	28	40	20
37	640	453	80	320	80	113	40
38	3620	1280	905	1280	453	453	160
38	28	20	10	10	5	10	5
39	453	320	113	226	40	80	20
40	226	320	113	226	57	57	28
41	905	226	160	453	160	113	40
42	1280	226	160	226	113	113	40
43	3620	1810	905	1810	1280	453	160
44	905	453	226	320	80	113	40
45	226	320	80	226	40	40	28

Table S8. VN_{50} values of sera collected from individuals with Omicron BA.1 breakthrough infection (mRNA-Vax^3 + BA.1)

Participant ID	VN_{50}						
	Wild-type	Alpha	Beta	Delta	Omicron BA.1	Omicron BA.2	Omicron BA.4
14	453	1280	1280	1280	640	905	226
15	453	640	640	640	453	453	80
16	1810	3620	1810	2560	1810	1280	453
17	453	1280	905	1280	453	640	160
18	453	640	640	640	640	320	57
19	640	1280	640	905	640	905	160
20	320	640	640	453	453	640	160
21	905	905	320	1280	320	453	80
22	320	453	453	640	640	453	80
23	1280	2560	1810	2560	1280	2560	640
24	5120	3620	3620	3620	1280	3620	640
25	5120	5120	5120	5120	5120	3620	453
26	160	113	40	160	40	113	7
27	1280	1280	1280	2560	905	905	320

Table S9. VN_{50} values of sera collected from individuals with Omicron BA.2 breakthrough infection (mRNA-Vax^3 + BA.2)

Participant ID	VN50						
	Wild-type	Alpha	Beta	Delta	Omicron BA.1	Omicron BA.2	Omicron BA.4
1	320	1810	320	453	226	320	113
2	3620	5120	3620	5120	3620	3620	905
3	320	905	226	226	160	226	113
4	160	226	113	113	80	226	40
5	905	2560	320	905	226	640	226
6	160	453	160	453	226	160	113
7	640	1810	320	640	640	640	226
8	640	1810	1810	1280	905	453	226
9	905	1810	2560	2560	640	1280	320
10	453	1810	320	453	226	320	160
11	320	1280	453	905	160	640	226
12	1280	2560	640	2560	320	1280	640
13	453	2560	1280	905	453	640	320
46	453	1280	453	905	113	640	226
47	453	2560	905	905	1280	640	226
48	2560	5120	2560	3620	640	1810	640
49	226	1810	113	320	226	453	113
50	640	1810	905	905	226	453	226
51	1810	2560	1810	3620	640	1810	320

.

Table S10. Vaccinated individuals analyzed for neutralizing antibody responses after depletion of RBD-/NTD-binding antibodies.

Characteristic	mRNA-Vax[3] + BA.2 (n=6)	mRNA-Vax[3] + BA.1 (n=6)	BNT162b2[3] (n=6)
Sex, n (%)			
Male	1 (17)	4 (67)	4 (67)
Female	5 (83)	2 (33)	2 (33)
Age, median (range)	41.5 (25-72)	32 (29-53)	36 (23-49)
Age group at vaccination, n (%)			
18-55 yrs	4 (67)	6 (100)	6 (100)
56-85 yrs	2 (33)	0 (0)	0 (0)
SARS-CoV-2 status, n (%)			
Positive	6 (100)*	6 (100)[#]	0 (0)
Negative	0 (0)	0 (0)	6 (100)†
Unknown	0 (0)	0 (0)	0 (0)
Interval, median (range)			
Days between D1/D2	28.5 (15-42)	39 (20-92)	‡
Days between D2/D3	230.5 (184-259)	189 (154-233)	219 (181-266)
Days until serum draw after D3	N/A	N/A	27.5 (26-30)
Days between last dose/infection	155 (129-200)	20 (3-66)	N/A
Days until serum draw after infection	40 (30-66)	43 (25-53)	N/A

N/A: not applicable; D, Dose; Yrs, Years; n, Number.

*, Individuals experienced SARS-CoV-2 breakthrough infections between March and May 2022, during which period the BA.2 lineage was dominant in Germany

[#], Omicron infection PCR-confirmed at time of recruitment to the research study. Individuals experienced SARS-CoV-2 breakthrough infections between November 2021 and January 2022, during which period the BA.1 lineage was dominant in Germany

†, No evidence of prior SARS-CoV-2 infection (based on COVID-19 symptoms/signs and SARS-CoV-2 PCR test)

‡, Participants received the primary 2-dose series of BNT162b2 vaccine as part of a governmental vaccination program and the interval between doses was not recorded.

Table S11. pVN$_{50}$ values of sera depleted of NTD or RBD-binding antibodies

Participant ID	Cohort	pVN$_{50}$											
		Wild-type			Omicron BA.1			Omicron BA.2			Omicron BA.4/5		
		Mock	NTD	RBD	Mock	NTD	RBD	Mock	NTD	RBD	Mock	NTD	RBD
7	mRNA-Vax^3+BA.2	1179	1021	200	996	1143	10	880	668	219	494	252	193
8	mRNA-Vax^3+BA.2	2039	1187	158	1276	1491	10	981	730	162	1148	525	123
9	mRNA-Vax^3+BA.2	2226	1607	673	1224	1276	10	1217	738	460	1139	420	535
11	mRNA-Vax^3+BA.2	1435	939	251	650	641	10	537	346	169	538	273	97
12	mRNA-Vax^3+BA.2	3562	2287	834	674	479	10	5562	805	1129	1797	626	1043
13	mRNA-Vax^3+BA.2	1463	2956	98	1401	1219	10	1403	732	247	821	634	118
15	mRNA-Vax^3+BA.1	1037	953	103	601	623	10	359	354	10	239	232	10
17	mRNA-Vax^3+BA.1	1401	1681	61	1525	1379	10	973	740	25	492	430	45
19	mRNA-Vax^3+BA.1	1992	1589	235	1754	1363	54	990	923	10	573	438	10
20	mRNA-Vax^3+BA.1	842	766	127	789	836	10	825	557	10	241	319	10
21	mRNA-Vax^3+BA.1	1404	1751	77	697	546	10	388	556	10	277	254	10
25	mRNA-Vax^3+BA.1	1870	1224	219	1439	1613	46	849	1216	27	886	612	10
30	BNT162b2^3	3818	2279	497	746	923	10	1349	644	463	620	238	278
32	BNT162b2^3	521	537	160	143	173	10	98	644	10	59	238	10
38	BNT162b2^3	2085	2332	348	1014	1147	39	844	931	38	364	276	56
43	BNT162b2^3	631	671	281	237	265	10	187	164	40	108	101	36
44	BNT162b2^3	1985	2071	463	745	591	10	525	648	61	247	222	47
45	BNT162b2^3	631	487	133	191	167	10	131	130	10	119	97	10
