# Supplementary material for: Omicron BA.2 breakthrough infection enhances cross-neutralization of BA.2.12.1 and BA.4/BA.5
Source: Sci Immunol. 2022 Sep 20:eade2283. doi: 10.1126/sciimmunol.ade2283 (PMC9529054; doi:10.1126/sciimmunol.ade2283)
Supplement: Supplementary file 2 — MDAR Reproducibility Checklist [file sciimmunol.ade2283_mdar_reproducibility_checklist.pdf]

Materials Design Analysis Reporting (MDAR)
Checklist for Authors

The MDAR framework establishes a minimum set of requirements in transparent reporting applicable to studies in the life sciences (see Statement of Task: doi:10.31222/osf.io/9sm4x.). The MDAR checklist is a tool for authors, editors, and others seeking to adopt the MDAR framework for transparent reporting in manuscripts and other outputs. Please refer to the MDAR Elaboration Document for additional context for the MDAR framework.

For all that apply, please note where in the manuscript the required information is provided.

Materials:

Newly created materials	indicate where provided: page no/section/legend)	n/a
The manuscript includes a dedicated "materials availability statement" providing transparent disclosure about availability of newly created materials including details on how materials can be accessed and describing any restrictions on access.	N/A: all materials were either purchased and are freely available, or was clinical trial	

Antibodies	indicate where provided: page no/section/legend)	n/a
For commercial reagents, provide supplier name, catalogue number and RRID, if available.	Materials and Methods – VSV-SARS-CoV-2 S variant pseudovirus generation / Depletion of RBD- or NTD-binding antibodies from human sera / Analysis of SARS-CoV-2-specific serum IgG (page 15, 18-19, Materials and Methods)<br><br>anti-VSV-G antibody (clone 8G5F11, Kerafast Inc.)<br>RBD-coupled magnetic beads (Acro Biosystems, MBS-K002<br>NTD-coupled magnetic beads (Acro Biosystems, MBS-K019)<br><br>SARS-CoV-2 panel multiplex ECLIA (Meso Scale Discovery, K15359U-2)	

DNA and RNA sequences	indicate where provided: page no/section/legend)	n/a
**Short novel DNA or RNA including primers, probes:** Sequences should be included or deposited in a public repository.		X

Cell materials	indicate where provided: page no/section/legend	n/a
**Cell lines:** Provide species information, strain. Provide accession number in repository **OR** supplier name, catalog number, clone number, **OR** RRID.	Materials and Methods – VSV-SARS-CoV-2 S variant pseudovirus generation / Live SARS-CoV-2 neutralization assay (pages 15-16 and 18, Materials and Methods):<br><br>HEK293T/17 (ATCC® CRL-11268™)<br>VERO 76 (ATCC® CRL-1587™)<br>Vero E6 (ATCC® CRL-1586™)	
**Primary cultures:** Provide species, strain, sex of origin, genetic modification status.		X

Experimental animals	indicate where provided: page no/section/legend)	n/a
**Laboratory animals or Model organisms:** Provide species, strain, sex, age, genetic modification status. Provide accession number in repository **OR** supplier name, catalog number, clone number, **OR** RRID.		X
**Animal observed in or captured from the field:** Provide species, sex, and age where possible.		X

Plants and microbes	indicate where provided: page no/section/legend)	n/a
**Plants:** provide species and strain, ecotype and cultivar where relevant, unique accession number if available, and source (including location for collected wild specimens).		X

**Microbes:** provide species and strain, unique accession number if available, and source.		X

Human research participants	indicate where provided: page no/section/legend) or state if these demographics were not collected	n/a
If collected and within the bounds of privacy constraints report on age, sex and gender or ethnicity for all study participants.	Materials and Methods – Study design and recruitment of participants and sample collection (pages 12-13):<br><br>Demographic and clinical information for all participants as well as sampling timepoints are provided in Tables S1-S3 and S10, and Fig. 1.	

Design:

Study protocol	indicate where provided: page no/section/legend)	n/a
If study protocol has been pre-registered, provide DOI. For clinical trials, provide the trial registration number **OR** cite DOI.	Trial NCT numbers are listed in M&M	

Laboratory protocol	indicate where provided: page no/section/legend)	n/a
Provide DOI **OR** other citation details if detailed step-by-step protocols are available.		X

Experimental study design (statistics details)		
**For in vivo studies:** State whether and how the following have been done	**indicate where provided: page no/section/legend. If it could have been done, but was not, write not done**	**n/a**
Sample size determination		X
Randomisation		X
Blinding		X
Inclusion/exclusion criteria		X

Sample definition and in-laboratory replication	indicate where provided: page no/section/legend	n/a
State number of times the experiment was replicated in laboratory.		
Define whether data describe technical or biological replicates.	Materials and Methods – Pages 13 - 20<br><br>Pseudovirus neutralization assay: n=2 technical replicates per serum per pseudovirus<br><br>Live SARS-CoV-2 neutralization assay: n=2 technical replicates per serum per virus	

Ethics	indicate where provided: page no/section/legend	n/a
**Studies involving human participants:** State details of authority granting ethics approval (IRB or equivalent committee(s), provide reference number for approval.		X
**Studies involving experimental animals:** State details of authority granting ethics approval (IRB or equivalent committee(s), provide reference number for approval.		X
**Studies involving specimen and field samples:** State if relevant permits obtained, provide details of authority approving study; if none were required, explain why.	Materials and Methods – Recruitment of participants and sample collection (pages 19- 20):<br><br>Individuals from the BNT162b2^3 cohort provided informed consent as part of their participation in the Phase 2 trial BNT162-17 (NCT05004181). Participants from the mRNA-Vax^3 + Omi BA.1 and mRNA-Vax^3 + BA.2 cohorts were recruited from University Hospital, Goethe University Frankfurt as part of a non-interventional study (protocol approved by the Ethics Board of the University Hospital [No. 2021-560]) researching patients that had experienced Omicron breakthrough infection	

	following vaccination for COVID-19.	

Dual Use Research of Concern (DURC)	indicate where provided: page no/section/legend	n/a
If study is subject to dual use research of concern regulations, state the authority granting approval and reference number for the regulatory approval.		X

Analysis:

Attrition	indicate where provided: page no/section/legend	n/a
Describe whether exclusion criteria were preestablished. Report if sample or data points were omitted from analysis. If yes report if this was due to attrition or intentional exclusion and provide justification.	Materials and Methods – Study design (page 13)<br><br>All participants had no documented history of SARS-CoV-2 infection prior to vaccination.	X

Statistics	indicate where provided: page no/section/legend	n/a
Describe statistical tests used and justify choice of tests.	Materials and Methods – Statistical analysis (page 20<br><br>The statistical method of aggregation used for the analysis of antibody titers is the geometric mean and for the ratio of SARS-CoV-2 VOC titer and wild-type strain titer the geometric mean and the corresponding 95% confidence interval. The use of the geometric mean accounts for the non-normal distribution of antibody titers, which span several orders of magnitude. The Friedman test with Dunn's correction for multiple comparisons was used to conduct pairwise signed-rank tests of group geometric mean neutralizing antibody titers with a common control group. The Kruskal-Wallis test with Dunn's correction for multiple comparisons was used to conduct unpaired signed-rank tests of group GMT ratios with a common control group. The Wilcoxon matched-pairs signed rank test was used for pairwise comparison the Omicron BA.4/5 neutralizing pVN50 titers with titers against the Omicron BA.1-BA.4/5 hybrid pseudovirus. Spearman correlation was used to evaluate the monotonic relationship between non-normally distributed datasets. All statistical analyses were performed using GraphPad Prism software version 9.	

Data availability	indicate where provided: page no/section/legend	n/a
For newly created and reused datasets, the manuscript includes a data availability statement that provides details for access or notes restrictions on access.	Section – Data and materials availability, page 30	
If newly created datasets are publicly available, provide accession number in repository **OR** DOI **OR** URL and licensing details where available.		X
If reused data is publicly available provide accession number in repository **OR** DOI **OR** URL, **OR** citation.		X

Code availability	indicate where provided: page no/section/legend	n/a
For all newly generated custom computer code/software/mathematical algorithm or re-used code essential for replicating the main findings of the study, the manuscript includes a data availability statement that provides details for access or notes restrictions.		X
If newly generated code is publicly available, provide accession number in repository, **OR** DOI **OR** URL and licensing details where available. State any restrictions on code availability or accessibility.		X

If reused code is publicly available provide accession number in repository **OR** DOI **OR** URL, **OR** citation.		X

Reporting

MDAR framework recommends adoption of discipline-specific guidelines, established and endorsed through community initiatives. Journals have their own policy about requiring specific guidelines and recommendations to complement MDAR.

**Adherence to community standards**	**indicate where provided: page no/section/legend**	**n/a**
State if relevant guidelines (e.g., ICMJE, MIBBI, ARRIVE) have been followed, and whether a checklist (e.g., CONSORT, PRISMA, ARRIVE) is provided with the manuscript.		X
